# The Face Inversion Effect Following Pitch and Yaw Rotations: Investigating the Boundaries of Holistic Processing

**DOI:** 10.3389/fpsyg.2012.00563

**Published:** 2012-12-18

**Authors:** Simone K. Favelle, Stephen Palmisano

**Affiliations:** ^1^School of Psychology, University of WollongongWollongong, NSW, Australia

**Keywords:** face recognition, inversion, holistic processing, pitch and yaw axes

## Abstract

Upright faces are thought to be processed holistically. However, the range of views within which holistic processing occurs is unknown. Recent research by McKone (2008) suggests that holistic processing occurs for all yaw-rotated face views (i.e., full-face through to profile). Here we examined whether holistic processing occurs for pitch, as well as yaw, rotated face views. In this face recognition experiment: (i) participants made same/different judgments about two sequentially presented faces (either both upright or both inverted); (ii) the test face was pitch/yaw rotated by between 0° and 75° from the encoding face (always a full-face view). Our logic was as follows: if a particular pitch/yaw-rotated face view is being processed holistically when upright, then this processing should be disrupted by inversion. Consistent with previous research, significant face inversion effects (FIEs) were found for all yaw-rotated views. However, while FIEs were found for pitch rotations up to 45°, none were observed for 75° pitch rotations (rotated either above or below the full face). We conclude that holistic processing does not occur for all views of upright faces (e.g., not for uncommon pitch rotated views), only those that can be matched to a generic global representation of a face.

## Introduction

By necessity, we must be able to generalize across many natural sources of image variation in order to recognize a face, including changes in distance and viewpoint as we move around each other and changes in lighting as we move in and out of different environments. We rarely view faces from directly in front (known as the “full face” or 0° view). Most commonly, our visual experience of faces falls within a range of viewpoints rotated away from 0° by up to 45° to the left or right (about the vertical axis; yaw), above or below (about the horizontal axis; pitch), and clockwise or anticlockwise (about the depth axis; roll). Within this range of variation in viewpoint, face recognition is remarkably good, even for unfamiliar faces (Favelle et al., 2011; Hill et al., 1997; Schyns and Bülthoff, 1994; Liu and Chaudhuri, 2002; Stephan and Caine, 2007). However, face recognition noticeably deteriorates for rotations greater than 45°. Face recognition performance for a yaw-rotated profile face view is poorer than that for both full face and yaw-rotated three-quarter (i.e., 45°) face views (Hill et al., 1997; Liu and Chaudhuri, 2002; McKone, 2008). Similarly roll and pitch rotations greater than 45° typically result in poorer face recognition performance than that for upright full faces (Favelle et al., 2007; Favelle et al., 2011; Martini et al., 2006; Valentine and Bruce, 1988). Why does face recognition become more difficult outside of this limited viewpoint range (0°–45°)?

Upright faces are recognized not as a collection of individual features, but as a “whole” percept. According to Rossion (2009), this holistic processing generates a “simultaneous perception of the multiple features of an individual face, which are integrated into a single global representation” (p. 305). Holistic processing is applied to all of the available cues to the face (both local ones arising from discrete features, as well as those based on the distances and spatial relationships between features), which are fused together into a single perceptual representation. If faces are both perceived and represented holistically then not only should the perception of a given facial feature depend on the perception of the face as a whole, but slight differences between faces should be discriminated very quickly and efficiently (at least under normal viewing conditions). While “configural processing” has often been used interchangeably with holistic processing (e.g., McKone, 2008), or as an umbrella term that includes holistic processing as a sub-type (e.g., Maurer et al., 2002), “configural information” is typically used to refer to the spacing or distance between the discrete features of a face (e.g., between the two eyes or between mouth and nose). The idea being that faces can be recognized based on differences in either configural (e.g., interocular distances) or featural (e.g., mouth surface reflectance or shape) information. For present purposes we shall use “holistic” to refer to the perceptual process and “configural information” to refer to the spatial distances and relationships between nameable facial features (e.g., Rossion, 2008).

A pillar of the face perception literature, the face inversion effect (FIE) is the observation that the inversion (180° roll or picture-plane rotation) of faces dramatically impairs recognition compared to upright faces, and that this impairment is disproportionately larger for faces than objects (Yin, 1969). Because the inversion manipulation preserves the low-level visual properties of the face present in the upright stimulus, the FIE can be attributed to high-level/cognitive processes used differentially for upright and inverted faces. Accordingly, inversion has been widely used as a control condition in behavioral studies (Valentine, 1988; Rossion and Gauthier, 2002; Rossion, 2008; Tanaka and Gordon, 2011; also see McKone et al., submitted).

It is now generally accepted that turning a face upside-down disrupts face-specific holistic processing. For example, if one creates a composite face by aligning the photographs of the top and bottom halves of two different faces, the obligatory holistic processing of the new “whole” face will impair naming accuracy and increase reaction times (RTs) for each half face (compared to when these half faces are misaligned – the “Composite effect”; Young et al., 1987). Similarly, studies have found that the memory for a facial feature is more accurate when it is subsequently presented in the context of the whole studied face than when it is presented on its own (the “Part-whole effect” – Tanaka and Farah, 1993). However, while both these Composite and Part-whole effects are strong for upright faces they disappear when the face stimuli are inverted (but see McKone et al., submitted).

Whether inversion produces a qualitative or a quantitative reduction in holistic face processing is currently a hotly debated topic (Richler et al., 2011; Rossion and Boremanse, 2008; Rossion, 2009). For example, based on their recent findings, Richler et al. (2011) claim that: (i) both upright and inverted faces are processed holistically; and (ii) the well-known FIE performance decrement arises because holistic processing is less efficient/successful for inverted faces (due to our limited experience with inverted faces). Other researchers argue that what is “lost” in an inverted face is the sensitivity to configural information and that featural information remains relatively unaffected (e.g., Carey, 1992; Freire et al., 2000; Maurer et al., 2002). However, when featural and configural information are equated for discriminability in upright faces, inversion appears to disrupt sensitivity to both types of information in a similar manner (McKone and Yovel, 2009; McKone and Robbins, 2011; Riesenhuber et al., 2004; Yovel and Kanwisher, 2004). These findings suggest that configural information may not always have a special status in face perception/recognition. In fact, according to Rossion (2009) all aspects of the face are “configural” when the face is being processed holistically. That is, the face has to be processed holistically to make the best use of both the available featural and configural information.

As noted above, the FIE is typically explained in terms of a disruption to holistic processing. It is possible to explain the poorer recognition for face views rotated more than 45° in pitch (from the full-face view) in a similar manner. During picture-plane rotations, Rossion and Boremanse (2008) found a non-linear drop in the holistic processing of faces (as measured with the composite face illusion) for roll rotations of 90° or more. They argued that the poor performance for faces at these unusual views (including the FIE) is based on the inability to match the incoming visual stimulus to an experience-derived holistic internal representation (i.e., a template) that is centered on the full-face view. Consistent with the idea that visual experience plays a key role in processing upright faces, Laguesse et al. (2012) found that adults trained to individuate a set of inverted faces showed a reduced FIE on a set of novel faces. If it is the case that holistic processing is reduced/impaired for views in the picture-plane with which we have less experience, this should also be the case for less experienced views rotated in the pitch and yaw axes.

To date there has been little investigation of holistic processing in faces rotated in pitch and yaw (as opposed to the extensive investigation of roll/picture-plane rotation on holistic processing, see Rossion and Boremanse, 2008). One exception is a study by McKone (2008) who examined performance with composite faces (made by aligning the half faces of two different individuals) rotated in yaw. She found that while identification of the individual face halves was poorer at profile views than full-face or three-quarter (45°) views, holistic processing was insensitive to view changes in yaw (as measured by both the “composite face” and “peripheral inversion” tasks – see McKone, 2004, 2008). These findings suggest that yaw viewpoint effects are driven by a disruption to featural processing. That is, profile views provide poor information about face parts but, despite the occlusion of half of the face, do provide adequate holistic information. In apparent conflict with the predictions of Rossion and Boremanse’s (2008) experience-only template theory outlined above, natural view frequency was found to have no effect on holistic processing in this study.

No studies have investigated holistic processing in pitch rotated views of faces, presumably because of the difficulty in applying the composite face task typically used to tap into this information. Favelle et al. (2011) used a scrambled/blurred paradigm to isolate the configural and featural information contained in faces following yaw, pitch, and roll rotations (up to 75° from the full-face view). They found that performance in a sequential face matching task based on configural information was best following roll camera rotations, poorer for yaw camera rotations, and poorer still for pitch camera rotations. While performance in this same task based on featural information was much poorer, it also showed similar patterns of viewpoint dependent decline in pitch and yaw, and no decline in roll camera rotations.

Two notable points arise from these findings. First, it appears that while both configural and featural information are utilized in recognizing faces across different views (at least views rotated up to 75°), configural information appears to be more useful. Second, pitch rotation disrupts configural information to a greater degree than yaw or roll rotation. Compared to rotations about other axes, pitch camera rotations result in a greater foreshortening and occlusion of features as well as a general reduction in the amount of available “face” information. Thus, Favelle et al.’s (2011) findings of a greater cost to face recognition following pitch camera rotations may be due to participants having to rely more heavily on parts- or object-based processing (as opposed to configural information and more face-specific, holistic processing) than in yaw. The aim of the experiment reported here is to investigate the idea that these viewpoint axis effects can be explained by differences in the degree to which parts/object-based or holistic processing is engaged.

**Figure 1:**
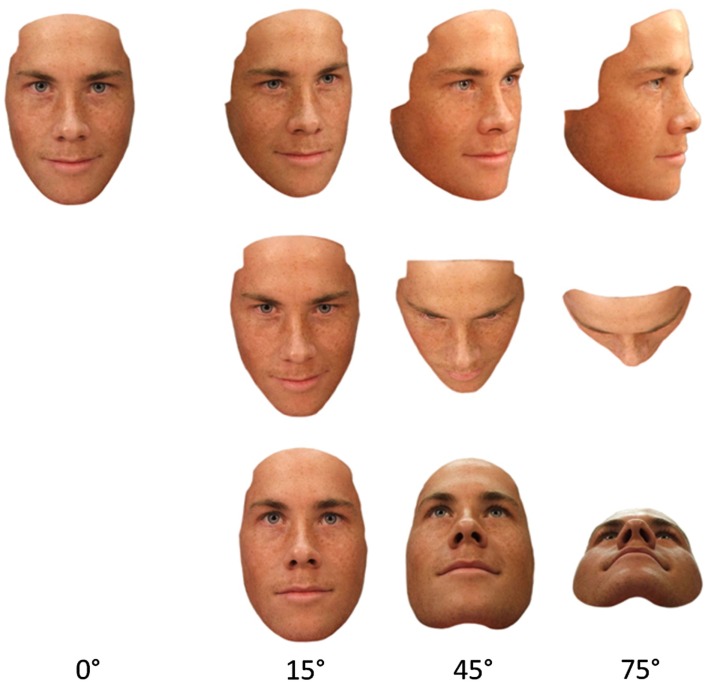
**Example of a set of the face stimuli**. Views are taken from rotations of 0°, 15°, 45°, and 75° in the yaw axis (top row), pitch axis above horizontal (middle row), and pitch axis below horizontal (bottom row). To view the stimuli inverted, rotate the page.

McKone (2008) found evidence for holistic processing in views of faces at 0°, 45°, and 90° of yaw rotation. Her study investigated face identification ability at different views (i.e., learning and testing at the same view) with the results suggesting that the functional role of holistic processing is to support reliable face identification across different images. Here we are considering the contribution of holistic processing to the transfer of learning across views (i.e., learning a face at one view and testing at another). Because of the difficulty in using a composite task for pitch rotated views, the current study used the FIE as an indicator of the disruption to holistic processing in faces (Rossion, 2008, 2009). Specifically we examined the FIE for matching unfamiliar, undistorted whole faces rotated in either yaw or pitch (see Figure 1). Views which contain sufficient “face” information to support holistic processing should generate a FIE. Thus, we expect to find FIEs for all yaw viewpoints (McKone, 2008; Favelle et al., 2011; Hills et al., 2012)^1^. However, based on previously observed face recognition difficulties with upright 75° pitch-up and -down rotated views, we may find little evidence of FIEs for these uncommon viewpoints. This would demonstrate (for the first time) that we are unable to access any holistic information contained in these particular pitch rotated face images.

## Materials and Methods

This study was approved by the University of Wollongong Human Research Ethics Committee, and written consent was obtained from all participants.

### Participants

Participants were 20 (six males) volunteer undergraduate students attending the University of Wollongong. The average age of participants was 21.6 years (age range: 18–29 years). All participants had normal or corrected-to-normal vision and none were familiar with the faces used as stimuli.

### Stimuli and design

Participants made “same/different” judgments of two sequentially presented faces, shown centrally, and both upright or inverted. On each trial, these two faces were typically seen from different views. There were 10 levels of viewpoint (0°, 15°, 45°, and 75° rotated left in yaw; 15°, 45°, and 75° rotated in pitch above; 15°, 45°, and 75° rotated in pitch below) crossed with orientation (upright and inverted), all manipulated within subjects. The dependent measures were matching sensitivity (*d*′, defined below) and RT to correct same matches.

The stimuli were high-quality digital facial photographs of nine Caucasian male models taken from 10 different viewpoints. Faces portrayed a neutral expression, and any distinctive features (such as moles or blemishes) as well as hair were removed digitally. Faces were illuminated by an ambient light source located directly above the model plus four directional light sources located 1 m in front of the model (just above and to the left, just above and to the right, just below and to the left, and just below and to the right of the face). The two lights located just above the face were oriented horizontally (i.e., at 90° to gravity), whereas the lights located just below the face were oriented 45° below the horizontal. Lighting was held constant across all viewpoints. In addition to a full-face (0°) view, each face was presented from three different viewpoints rotated 15°, 45°, and 75° away from 0° in three different directions: yaw left^2^, pitch-up (above) and pitch-down (below). There were 10 different viewpoint images generated for each face, and each of these was also inverted (rotated 180° in the picture plane), resulting in 20 different images per face. As these image manipulations were implemented to each of the nine face stimuli, overall there were 180 images created for the task (see Figure 1)^3^.

All images were viewed in the center of the computer screen against a white background. The on-screen height of 0° faces was approximately 16 cm with a width of 10 cm, which produced a visual angle of 14.7° × 19.2°. For yaw viewpoints the height of the face image remained constant, however face width increased as the viewpoint was rotated further away from 0°. Face width remained constant for pitch camera rotations, however face height decreased as the viewpoint was rotated further away from 0° (for both pitch-up and pitch-down conditions). The 75° pitch-up camera condition had the shortest image, which was 12 cm high and produced a visual angle of 11° × 9.2°. The rectangular patterned mask used in the experiment subtended a visual area of 18° × 22° and was composed of various elements taken from the stimuli used in the task.

### Apparatus

Full color images were presented to participants on a 48 cm flat-screen monitor with a resolution of 1024 × 768 pixels. Trials were run on a Macintosh G4 computer and RSVP experimental software (Version 4.0.5; www.tarrlab.org) guided the trial sequence. Responses were made via key presses on a keyboard placed in front of the participant.

### Procedure

Participants completed the experiment in a dimly lit room in undisturbed conditions. The task was a sequential matching task in which an initial face (face 1, viewed at full-face 0°) was presented first followed by a mask, and then a test face (face 2, viewed at any of the 10 possible viewpoints) was presented followed by the mask again. Participants were first verbally instructed how to complete the task, with emphasis placed on both speed and accuracy in responding. Written instructions on how to complete the task were also provided on the computer screen. After reading the instructions participants completed 10 practice trials to familiarize them with the task. Stimuli used in the practice trials were different from the stimuli used in the task. Following the practice trials participants were given a chance to ask any questions about the procedure, should they have any, before continuing on with the experiment.

Orientation (upright or inverted) was blocked and the order of the blocks counterbalanced. Each block consisted of 180 trials, giving a total of 360 experimental trials. In half of the trials the two faces presented were the same, regardless of viewpoint (same trials). In the other half of trials the two faces were different; the different face was randomly selected from the remaining eight faces (different trials). Trial type was presented in random order within each block. Participants were given five self-timed rest periods spaced equally throughout each block. The experiment lasted approximately 30 min.

Each trial began with a fixation cross displayed for 500 ms. This was followed by the presentation of face 1 for 250 ms. Then the mask was presented for 500 ms. Face 2 was then presented for 250 ms, followed by a second presentation of the mask for 500 ms. Following the second mask the screen remained blank for 2 s or until a response was made by the participant. If a response was not made within this time, the trial ended (i.e., “timed-out”). The interval between trials was 1 s. Participants were required to respond by pressing clearly labeled “same” and “different” keys on a keyboard depending on whether they judged face 1 and face 2 to be the same face or two different faces.

## Results

Participants’ responses were converted into hit (H) and false alarm (FA) rates, where a hit was a correct “same” response to a face, and a FA was an incorrect “same” response to a “different” face. These H and FA rates were converted into *z*-scores and then used to calculate *d*′ (see MacMillan and Creelman, 1991). The RT data was based on participants’ correct responses to only the same trials. Trials that timed-out were not included in the analysis (timed-out trials accounted for 0.6% of total trials). Mean sensitivity (*d*′) and RT (ms) scores were analyzed in a series of two-way (viewpoint × orientation) repeated measures ANOVAs. Pitch viewpoints (seven levels: −75°, −45°, −15°, 0°, +15°, +45°, and +75°) were analyzed separately to yaw viewpoints (four levels: 0°, 15°, 45°, and 75°). The alpha level was 0.05. Greenhouse–Geisser corrections were made whenever the assumption of sphericity was violated. Bonferroni adjustments were made where necessary to control for family wise error.

### Analysis of data from pitch viewpoints

#### Signal detection analysis

**Figure 2:**
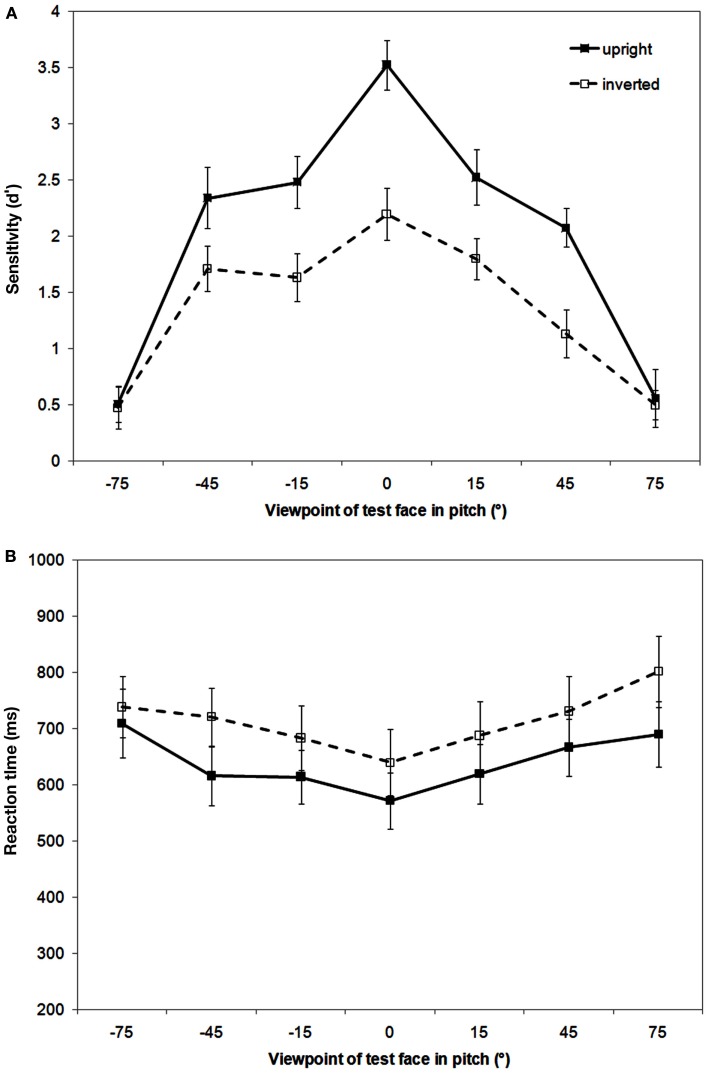
**Results of the sequential matching task showing mean recognition sensitivity [*d*′ (A)] and reaction time [ms (B)] as a function of pitch viewpoint (where – refers to viewpoints below 0° and +refers to viewpoints above 0°) and orientation (upright and inverted)**. Error bars represent 1 SEM.

As can be seen in Figure 2 (top panel), sensitivity to matching faces is viewpoint dependent with sensitivity highest for full-face views and declining with increased rotation in either direction away from this view. A repeated measures ANOVA conducted on *d*′ data revealed a main effect of orientation, *F*(1,19) = 25.75, *p* < 0.001, MSE = 30.12, $\eta_{p}^{2}$ = 0.58, with sensitivity to matching inverted faces lower than upright faces, and a main effect of viewpoint, *F*(6,114) = 43.07, *p* < 0.001, MSE = 30.85, $\eta_{p}^{2}$ = 0.69. These main effects are qualified by a significant interaction between orientation and viewpoint, *F*(6,114) = 3.17, *p* < 0.01, MSE = 2.19, $\eta_{p}^{2}$ = 0.14. *Post hoc* comparisons showed a significant FIE (upright–inverted) at the full-face (0°) view and at pitch viewpoints of 15° and 45° above and below 0° (all *p* < 0.05) with no evidence of a FIE at either of the 75° viewpoints (both *p* > 0.84).

#### RT data analysis

The pattern of the RT data approximately reflected that of the sensitivity data except at pitch +75° (see Figure 2, bottom panel). The ANOVA revealed a main effect of orientation, *F*(1,19) = 4.77, *p* < 0.05, MSE = 381809.7, $\eta_{p}^{2}$ = 0.20, with significantly faster response times to upright than to inverted faces, and of viewpoint, *F*(6,114) = 8.15, *p* < 0.001, MSE = 156663.29, $\eta_{p}^{2}$ = 0.30. There was no interaction between orientation and viewpoint, *F*(6,114) = 0.74, *p* = 0.56, MSE = 12112.8. *Post hoc* comparisons showed significantly faster response times to 0° than to either 45° or 75° pitch viewpoints above (both *p* < 0.01).

### Analysis of data from yaw viewpoints

#### Signal detection analysis

**Figure 3:**
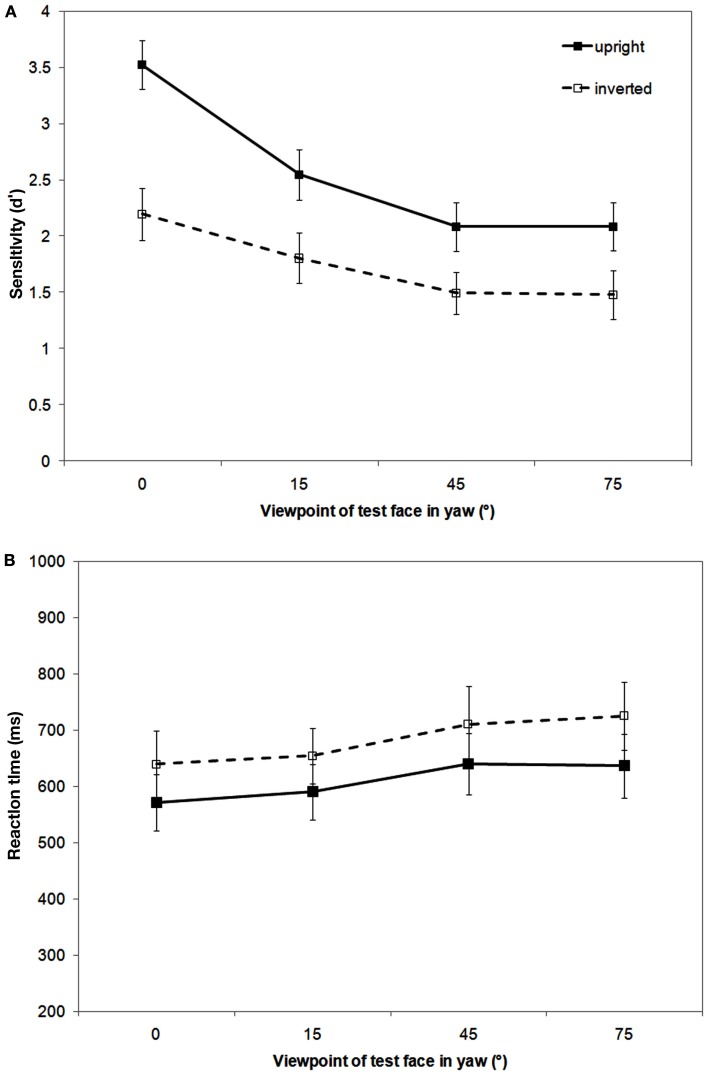
**Results of the sequential matching task showing mean recognition sensitivity [*d*′ (A)] and reaction time [ms (B)] as a function of yaw viewpoint and orientation (upright and inverted)**. Error bars represent 1 SEM.

As can be seen in Figure 3 (top panel), sensitivity to matching faces is viewpoint dependent with sensitivity highest for full-face views and declining with increased rotation away from this view. A FIE is apparent at all yaw viewpoints. A repeated measures ANOVA conducted on sensitivity data revealed a main effect of orientation, *F*(1,19) = 39.76, *p* < 0.001, MSE = 26.71, $\eta_{p}^{2}$ = 0.68, with sensitivity higher to upright than inverted faces, and a main effect of viewpoint, *F*(3,57) = 15.73, *p* < 0.001, MSE = 10.28, $\eta_{p}^{2}$ = 0.45, with no interaction between them, *F*(3,57) = 1.86, *p* = 0.15. *Post hoc* comparisons showed significantly higher sensitivity to 0° viewpoints than to any other yaw viewpoint (all *p* < 0.03). There was no difference in sensitivity to matching faces between 15°, 45°, and 75° viewpoints (all *p* > 0.16).

#### RT data analysis

The RT data reflects the pattern of the sensitivity data with a FIE evident at all viewpoints (see Figure 3, bottom panel). A repeated measures ANOVA showed significantly faster responses to upright than inverted faces, *F*(1,19) = 4.8, *p* < 0.05, MSE = 212161.9, $\eta_{p}^{2}$ = 0.20, and a main effect of viewpoint, *F*(3,57) = 5.98, *p* < 0.001, MSE = 56426.8, $\eta_{p}^{2}$ = 0.24, with no interaction between them, *F*(3,57) = 0.16, *p* = 0.93. *Post hoc* comparisons showed significantly faster responses to 0° viewpoints than to either 45° or 75° yaw viewpoints (both *p* < 0.05).

## Discussion

As in previous experiments from this lab (Favelle et al., 2007, 2011), the recognition of upright faces from photographic images was found to be viewpoint dependent following both pitch and yaw rotations. Here performance was measured in terms of face recognition sensitivity (*d*′). We found that recognition performance was most sensitive for the upright full-face views and declined as these upright faces were rotated away from the full-face view. While sensitivity continued to decline for upright face views following pitch rotations of up to ±75° (i.e., above/below the upright full-face view), no further decline was evident following yaw rotation beyond 45°.

Interestingly, inversion impaired face recognition sensitivity (i.e., the FIE) for some viewpoints, but not for others. As Hills et al. (2012) found, recognition sensitivity for all yaw-rotated faces was clearly disrupted by inversion. However, while recognition sensitivity for faces rotated up to ±45° in pitch was also vulnerable to inversion, there was no evidence of any FIE for ±75° pitch rotated views. These 75° pitch rotated conditions had the lowest recognition sensitivities of all the viewpoints tested. However, given that the *d*′ values for these conditions were still around 0.5 (which corresponds to a proportion correct of ∼0.6) the absence of inversion effects for these viewpoints was unlikely to have arisen due to floor effects.

The advantage of the face inversion manipulation is that the low-level stimulus properties present in an upright face are identical to those in an inverted face. Thus, any differences between the two conditions have to be accounted for by higher-level, probably face-specific, processes. As noted in the Section “Introduction,” the best candidate for this appears to be holistic processing (Rossion, 2008, 2009). The results of the present experiment suggest that holistic processes are called on only within a certain range of viewpoints of an upright face. Our finding that faces rotated to all yaw viewpoints were vulnerable to inversion is in line with Hills et al. (2012) and also with McKone (2008), who found evidence for holistic processing at 0°, 45°, and 90° yaw views using composite and peripheral inversion tasks. Interestingly, while recognition sensitivity declined for both upright and inverted faces as yaw rotation increased from 0° to 45°, the size of the FIE did not vary significantly across the different yaw viewpoints (as indicated by the lack of interaction between orientation and viewpoint. Also see Van der Linde and Watson (2010) for evidence of quantitative decline in yaw and roll rotations). These findings suggest that: (i) the gradual viewpoint dependent declines in recognition sensitivity were driven by the loss of featural information and subsequent changes to configural information; whereas (ii) the relatively constant decrement in sensitivity produced by inversion was the consequence of the disruption to holistic processing (McKone, 2008; Favelle et al., 2011).

The current experiment is the first to investigate the holistic processing of pitch rotated face views. While the FIE was observed at pitch ±15° and ±45° viewpoints, the absence of a FIE at pitch ±75° viewpoints suggests that there is a fixed limit to the range of upright face views in which holistic processing occurs. That is, there appears to be a qualitative difference in the way that upright faces are processed at pitch ±75° viewpoints compared to the other pitch and yaw-rotated viewpoints tested in this experiment. Face recognition performance at these viewpoints could be explained in two ways: (i) holistic processing occurs much less efficiently at 75° pitch rotations because we have little to no expertise with these views (Richler et al., 2011), or (ii) there was insufficient face information for holistic processing to occur for 75° views.

Recently, Richler et al. (2011) have argued that holistic processing is applied less efficiently for faces at views with which have less expertise. We do have little experience with pitch 75° views, however, they will occur from time to time in everyday life (e.g., when lying on the ground looking up, or when up on a ladder looking down, say at a person standing on the ground). But presumably we have even less expertise with inverted pitch 75° views. So rather than leading to the observed qualitative difference in face recognition sensitivity, we might expect these views to lead to an, albeit large, but still quantitative reduction in sensitivity relative to more common views. Further, performance in the current study is measured with (*d*′) and hence cannot be explained in terms of response bias^4^. While the view expertise account cannot fully explain the current results, future studies in which participants were extensively trained to recognize faces from uncommon views (i.e., rotated 75° above or below the full-face view in the pitch axis) could test this proposal.

Rossion and his colleagues (Rossion, 2008, 2009; Rossion and Boremanse, 2008; Van Belle et al., 2010) on the other hand argue that our visual experience should produce qualitative differences in our recognition sensitivity for upright and inverted faces. They propose that: (i) based on our extensive visual experience with upright faces, our face recognition system has developed a generic global representation of a face (i.e., based on a template or possibly templates)^5^; and (ii) following inversion, the incoming visual face stimulus can no longer be matched to this template/s. As a result, the inverted face image cannot be processed holistically and instead has to be analyzed sequentially at the level of local parts/features/elements. In support of this idea, Rossion and Boremanse (2008) found a non-linear shift in holistic processing of faces (in a composite face task) following picture-plane rotations past 90°.

More recently, Van Belle et al. (2010) found empirical evidence that face inversion constricts the observer’s functional visual field. Their research suggests that: (i) when viewing an upright face, an observer’s perceptual field encompasses the entire face, which allows him/her to bind all the available face information into a holistic representation; and (ii) when viewing an inverted face, this perceptual field is narrowed to a smaller spatial window (e.g., the eye region). Processing of information within this field is preserved, whereas information outside the perceptual field, such as long-range distances or multi-element relationships, is lost.

Following on from this, the current results might be accounted for in the same way. According to this view, all faces (at least all faces rotated in the picture-plane) are handled in a similar way by the early visual system. However, it may not be possible to match 75° pitch rotated views of faces to the experience-derived face template/s and so as a result they do not appear to initiate holistic processing. If this is the case, since neither 75° pitch rotated views of faces nor any inverted faces are processed holistically, then there is no basis for the FIE. Importantly, what this suggests is that a visual stimulus simply being categorized as a face, as we assume at least the 75° pitch view from below would, is not enough to trigger holistic processing.

## Conclusion

Changes in view can result in substantial variations in the visual appearance of a face. Despite this, our results show that holistic processing occurs for a large range of views around the upright full-face view. Specifically, the range of views within which upright faces are processed holistically extends to rotations of 90° in yaw (McKone, 2008) and ±45° in pitch. For views rotated beyond 90° in roll (Rossion and Boremanse, 2008) and 45° in pitch, there appears to be a qualitative shift in performance. It would appear that when views of faces are inverted or rotated such that a significant amount of face information is lost (e.g., by 75° in pitch), recognition must rely on some other type of processing. In principle, in these situations faces might be processed (i) holistically, but very inefficiently (Richler et al., 2011), (ii) as faces but in a non-holistic manner (Rossion, 2009), or (iii) as an object (i.e., object processing may not be the same thing as processing a face analytically, feature-by-feature). The nature of this processing is up for debate and is a topic worthy of future research.

## Conflict of Interest Statement

The authors declare that the research was conducted in the absence of any commercial or financial relationships that could be construed as a potential conflict of interest.

## Footnotes

^1^Van der Linde and Watson (2010) previously examined face recognition for views rotated up to 180° in the roll and yaw axes simultaneously. In both same view (Experiment 1) and different view (Experiment 2) sequential matching tasks they found evidence of viewpoint dependent declines in performance following yaw and roll rotations with no interaction between the two axes. Based on this, they also argue that the shift from holistic to feature-based processing in roll and yaw is quantitative, and not qualitative.

^2^The face images were of real people and captured using a digital camera mounted on a semi-circular frame (see Favelle et al., 2007) so to minimize noise (e.g., slight variations in camera angle or model expression) only one direction of yaw was captured. Since the primary function of the yaw viewpoints was to ensure a replication of the FIE as suggested by the findings of McKone (2008) and found by Hills et al. (2012) and to provide a frame of reference for the FIE in pitch, only one direction of yaw rotation was included.

^3^We appreciate that nine different face identities is not large and that it is possible participants become familiar with the faces, however, earlier work with faces from this database shows a similar viewpoint dependent pattern for both sequential matching and old/new recognition tasks (Favelle et al., 2007). Since each face is viewed the same number of times in our experiment, any familiarity gained would not have a systematic effect on performance.

^4^Separate analyses of response bias (*c*) show no influence of inversion on response bias for either pitch, *F*(6,114) = 0.95, *p* = 0.46, or yaw views, *F*(3,57) = 1.4, *p* = 0.25. That is, there is no interaction between viewpoint and orientation in response bias measure.

^5^McKone (2008) notes that the internal representation of the global structure of the face could be determined by either experience-only (as appears to be suggested by Rossion and his colleagues) or by a combination of an innate expectation for upright faces and our visual experience. Since she has found that holistic information is unaffected by yaw rotation, she proposes that either the template in question is 3D or that there are multiple templates (all upright) each coding different yaw viewpoints. This type of explanation can be extended to the current findings for pitch viewpoints, where either the 3D template might only be a partial representation or the set of templates do not code for pitch rotated viewpoints beyond 45°.
